# Ophthalmic manifestations of nasopharyngeal carcinoma (NPC): A systematic review of reported cases with and without prior history of nasopharyngeal carcinoma

**DOI:** 10.5935/0004-2749.2022-0241

**Published:** 2024-02-23

**Authors:** Hind Manaa Alkatan, Saleh Hamad Alrashed, Azza MY Maktabi

**Affiliations:** 1 Department of Ophthalmology, College of Medicine, King Saud University, Riyadh, Saudi Arabia; 2 Department of Pathology and Laboratory Medicine, College of Medicine, King Saud University, Riyadh, Saudi Arabia; 3 King Saud University Medical City, King Saud University, Riyadh, Saudi Arabia; 4 Pathology and Laboratory Medicine Department, King Khaled Eye Specialist Hospital, Riyadh, Saudi Arabia

**Keywords:** Nasopharyngeal carcinoma, Carcinoma, Eye manifestations, Exophthalmos, Diplopia, Systematic review

## Abstract

**Purpose:**

We aimed to study reported cases of nasopharyngeal carcinoma presenting with ophthalmic manifestations with and without a prior diagnosis of nasopharyngeal carcinoma.

**Methods:**

We conducted a systematic review following the Preferred Reporting Items for Systematic Reviews and Meta-Analyses (PRISMA). A literature search was conducted using the MEDLINE database in PubMed and Google Scholar. We included patients with a previous diagnosis of nasopharyngeal carcinoma in Group I and those without a prior diagnosis of nasopharyngeal carcinoma in Group II. Data included demographics, clinical presentation, history of nasopharyngeal carcinoma, treatment, histopathological description, World Health Organization classification, and outcome.

**Results:**

Fifty-eight patients (26 in Group I and 32 in Group II) were included. The male-to-female ratio was 3:1. The mean age of the patients (53.3 ± 11.7 years and 54.8 ± 16.2 years, respectively) and gender did not differ significantly between the two groups. The most common ocular presentations were diplopia and proptosis in the first group (each in 34.6%), whereas visual disturbance was most common in the second group (46.9%). Treatment options and World Health Organization grading were comparable. The outcome in 38 patients (after a comparable follow-up period) was significantly better in group II (p=0.003). There was no statistically significant difference in the outcome of 23 patients in correlation with World Health Organization grades II versus III irrespective of group (p=0.094).

**Conclusions:**

The demographics of patients with nasopharyngeal carcinoma presenting with ophthalmic manifestations were similar between the two study groups, with a wide age range and male predominance. Patients presenting initially to ophthalmologists with no history of nasopharyngeal carcinoma have a more favorable outcome. World Health Organization grading may have less value as a prognostic indicator.

## INTRODUCTION

Nasopharyngeal carcinoma (NPC) is the most common tumor in this location and occurs particularly frequently in the Chinese population. The etiology of NPC has been linked to environmental factors such as smoking, chemical fumes, volatile agents, and the use of herbal medications and/or nasal oils^(1)^. Oncogenesis has also been suggested to play a role in NPC pathogenesis due to the high prevalence of Epstein-Barr virus (EBV) in these cases, and researchers have also advocated for inclusion of human leukocyte antigens in the etiology and prognosis of NPC^(1)^. The tumor is also known as *lymphoepithelioma and lymphoepithelial carcinoma* due to the observed prominent lymphoid component in these tumors. A definitive diagnosis of NPC by positive biopsy is required not only for the initiation of proper therapy but also for histopathological classification. The clinical presentation of NPC is variable and can be primarily nasal or otologic, and it also includes the presence of a neck mass or cranial nerve palsy. The latter occurs due to the extension of the tumor superiorly, causing skull base erosion manifesting with headache, facial pain, and diplopia^(2)^. When there is an orbital involvement, particularly in recurrent NPC, ophthalmologists might be the first to diagnose this tumor^(3)^. The orbital involvement in NPC is relatively rare and is classified as stage T4 disease^(4)^. We have previously described a case series of NPC patients diagnosed initially by an ophthalmologist due to the initial ocular presentation of the tumor without a history of NPC^(5)^. In that series with orbital involvement, the 3-year survival rate of the patients with orbital involvement due to recurrent NPC was only 49%^(4)^. However, all of our cases were considered as primary NPC without a history of the disease, and no definite survival rate was calculated considering the number of cases included^(1)^.

In this systematic review, we aim to study all previously reported cases of NPC presenting with ophthalmic manifestations with and without a prior diagnosis of NPC as participants and to compare the two groups to determine if there are any significant differences in the demographics, clinical presentation, histopathological grading, or outcomes despite variable therapy in this unique initial ophthalmic presentation in the group with no prior diagnosis of NPC. We used search tools focusing on the population/problem, intervention/indicator, comparison/control, and outcomes (PICOS)^(6)^. We followed the updated World Health Organization (WHO) 2017 classification for head and neck tumors: well-differentiated keratinizing squamous cell carcinoma as type I, nonkeratinizing (with differentiated and undifferentiated types) as type II, and basaloid squamous cell carcinoma as type III^(7)^.

## METHODS

This systematic review was approved by the Human Ethics Committee/Institutional Review Board at King Khaled Eye Specialist Hospital (RP-2062) and was conducted in compliance with the Declarations of Helsinki. To ensure the quality of our review, we used the Preferred Reporting Items for Systematic Reviews and Metanalyses (PRISMA) statement in our methodology^(8)^.

### Data acquisition

**Figure 1:**
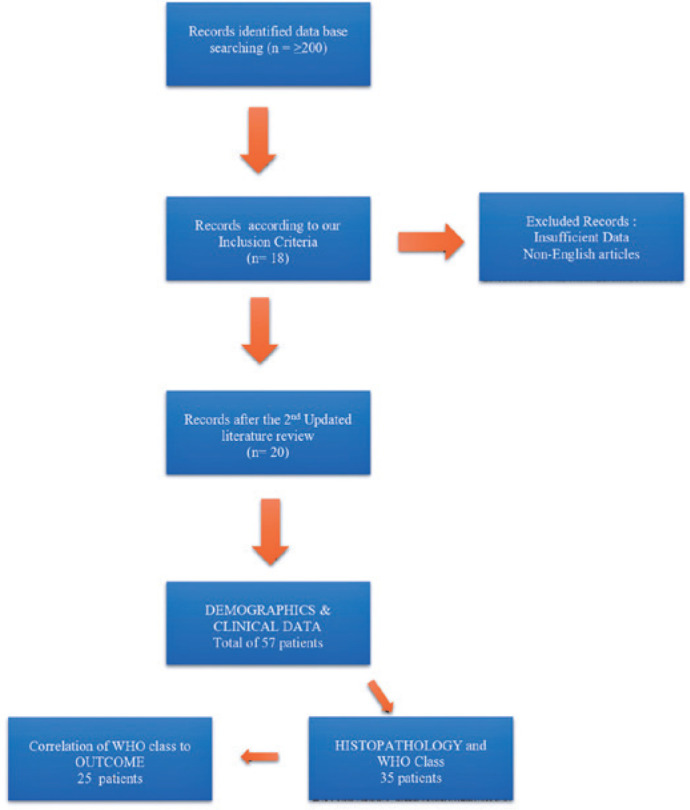
Flow diagram indicating the process of our literature search, data collection, and management.

We performed a literature search twice, on two separate occasions in 2020 and 2021, using the MEDLINE database in PubMed and Google Scholar. We used the following words in the search: nasopharyngeal carcinoma/NPC and ophthalmic presentation OR NPC and orbit OR NPC and decreased vision OR NPC with cranial palsy. Each paper was carefully reviewed to extract those with any documented ophthalmic presentation. Inclusion criteria were all available published NPC cases in the literature with ophthalmic presentation, and we included patients who had a previous history/diagnosis of NPC with ophthalmic presentation as Group I and those with ophthalmic symptoms as their initial primary presentation before their diagnosis of NPC as Group II. We aimed to include the following data: demographic information, clinical presentation, history or no history of NPC diagnosis, modality of treatment, histopathological description, WHO classification, and outcome following treatment (dead or alive). Exclusion criteria were non-English-written reported cases and papers with insufficient data reporting. With regard to the WHO classification, histopathology was categorized as WHO I, WHO II, and WHO II according to the latest classification. Papers that included detailed histopathological descriptions sufficient for classifying these were included, and the classification was entered in the collected data sheet. We excluded articles with deficient reported information that was required and essential for our meta-analysis. Thus, after excluding papers that lacked a histopathological description, we also filtered out papers that did not include the outcome, because we aimed to compare and analyze the outcome in relation to the WHO classification. All papers that were gathered were either case series or case reports. A total of 20 articles were gathered, which included 57 patients^(1,3,9-26)^. These were analyzed in the first part of our study. Only 35 patients of the gathered articles had a clear histopathological description; of those, only 25 patients had documented outcomes, and these patients were analyzed in the second stage of our study. Figure 1 shows a flowchart indicating the process of our literature search and data collection and management.

### Statistical analysis

We gathered all available patient demographic information, choice of treatment, follow-up duration, and patient life status at the end of follow-up in a Microsoft Excel sheet. The collected cases were then divided into two well-defined groups: the first group included cases of NPC with prior known history and/or diagnosis of NPC and yet presenting with ocular presentation, and the second group included cases of NPC presenting with ocular symptoms initially with no history nor previously diagnosed NPC. All available data were entered into a Microsoft Excel sheet.

Data were analyzed using SPSS^®^ version 21.0 (IBM Inc., Chicago, IL, USA). We performed descriptive analysis, in which categorical variables were presented in the form of frequencies and percentages and continuous variables in the form of mean (± standard deviation) and range (minimum to maximum). We used an independent *t*-test to compare the means between the two groups and the chi-square test to compare proportions between the groups. Any output with a p-value less than 0.05 was interpreted as an indicator of statistical significance.

## RESULTS

**Figure 2:**
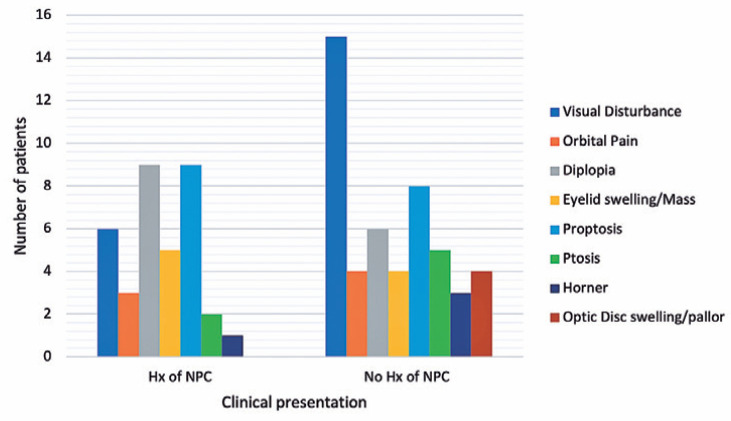
Demonstration of the clinical presentation in the two groups with and without a known history (Hx) of nasopharyngeal carcinoma (NPC).

Of the 20 articles, 58 patients were gathered, including 44 men and 14 women with a mean age of 54.8 ± 16.2 years. Of these, 26 patients had a diagnosis of NPC before the ophthalmic presentation (Group I) and 32 had an initial ophthalmic presentation leading to the diagnosis of NPC (Group II). Table 1 presents a summary of the results of these 58 patients with a comparison between the two groups. There was no statistically significant difference in age at presentation or gender distribution between the two groups. The most common clinical presentation in group I patients with a history of NPC was diplopia and proptosis in 34.6%. In contrast, almost half of the patients (47%) in group II complained of visual disturbance, followed by proptosis in one-quarter of patients (25%). Figure 2 demonstrates the clinical presentation in the two groups.

**Table 1:** Comparison of the demographics and clinical presentation of 58 patients with NPC who had ophthalmic manifestations with and without previous diagnosis of NPC

Characteristic	Group I: With history of diagnosed NPC (n=26)	Group II: Without history of NPC (n=32)	p-value
**Age**			
Age in years - all, mean ±SD [ Range]	53.3 ± 11.7 [22-74]	54.8 ± 16.2 [27-92]	0.699
Age in years men, mean ±SD [ Range]	54.1 ± 11.4 [22-70]	54.3 ± 16.2 [27-92]	0.948
Age in years women, mean ±SD [ Range]	50.8 ± 13.2 [37-74]	56.1 ± 17.1 [30-78]	0.542
**Gender**			
Male (n=44)	20 (76.9)	24 (75.0)	0.865
Female (n=14)	6 (23.1)	8 (25.0)	
**Clinical Presentation**			
Visual Disturbance	6 (23.1)	15 (46.9)	0.063
Orbital Pain	3 (11.5)	4 (12.5)	0.908
Diplopia	9 (34.6)	6 (18.8)	0.176
Eyelid swelling/Mass	5 (19.2)	4 (12.5)	0.487
Proptosis	9 (34.6)	8 (25.0)	0.428
Ptosis	2 (7.7)	5 (15.6)	0.362
Horner	1 (3.8)	3 (9.4)	0.406
Optic Disc swelling/pallor	0 (0.0)	4 (12.5)	0.064

With regard to treatment, 25 patients received chemotherapy, 44 were treated with radiotherapy, 30 received a combined treatment, and 5 underwent surgery only. Table 2 demonstrates the distribution of treatment modalities between the two groups. Most patients were treated by chemotherapy, radiotherapy, or both, and there was no statistically significant difference in the frequency of the chosen method of treatment between the two groups. Orbitotomy was infrequently used in 11.5% in Group I and 6.25% in Group II. With regard to the outcome, we were unable to extract and correlate the treatment modality with survival due to an inconsistency in reporting and the lack of information in some reports. Of the 58 patients, only 38 had clearly documented outcomes over a variable period of follow-up ranging from 1 month to a maximum of 180 months in both groups combined, with an average of 24.1 ± 17.2 months in the first group and 26.9 ± 38.8 months in the second group. In the 17 patients previously diagnosed with NPC, 70.6% had poor outcomes (disease-related death), whereas of the 21 patients who were diagnosed with NPC for the first time when presenting to an ophthalmologist, 81% were alive. This was found to be statistically significant, with a p-value of 0.003 at 5% significance.

**Table 2:** Comparison of the treatment modalities in 58 NPC patients between those who were diagnosed prior to their ocular presentation and those who presented initially to the ophthalmic service and were diagnosed after their presentation†

Characteristic	Group I: With history of diagnosed NPC (n=26)	Group II: Without history of NPC (n=32)	p-value
**Treatment**			
Chemotherapy (n=35)	18 (69.2)	17 (53.1)	0.212
Radiotherapy (n=44)	22 (84.6)	22 (68.8)	0.160
Chemotherapy and radiotherapy (n=30)	16 (61.5)	14 (43.8)	0.178
Orbitotomy (n=5)	3 (11.5)	2 (6.2)	0.475
**Follow-up** in months, mean ±SD [ Range]	24.1 ± 17.2 [2-64]	26.9 ± 38.8 [1-180]	0.786
**Outcome** (n=38) ^†^			
Alive (n=22)	5 (29.4)	17 (81.0)	0.003^*^
Dead (n=16)	12 (70.6)	4 (19.0)	0.908

* Statistically significant at the 5% level of significance.

† Note that the outcome was compared between the two groups in a total of 38 patients (17 in group I and 21 in group II).

**Figure 3:**
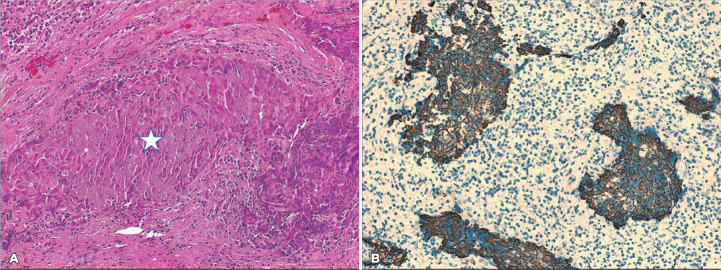
(A) Example of WHO grade II NPC with nonkeratinizing islands of squamous cell carcinoma (white star) and surrounding chronic inflammation (original magnification ×200 hematoxylin and eosin). (B) The areas of tumor expressing a reaction to Pan-cytokeratin immunohistochemical marker (original magnification ×200 CytoK).

In the second part of our analysis, based on the histopathological classification and comparison according to the WHO grading, we initially compared the WHO grading between the same two groups in 35 patients, and more than half of the patients in both groups were classified as WHO III (55.6% and 57.7%, respectively) with no statistically significant difference (Table 3). The outcome was available for only 24 of the 35 patients, and when we correlated the outcome of 23 of 24 patients (1 patient with WHO grade I was excluded) in relation to WHO grades II (Figure 3) versus III irrespective of the group to which they belong in Table 4, there was no statistically significant difference in the outcome (p=0.094).

**Table 3:** Comparison of the WHO grading in 35 NPC patients who had ophthalmic manifestations between 2 groups: those who were diagnosed prior to their ocular presentation and those who presented initially to the ophthalmic service and were diagnosed after their presentation

Characteristic	Group I: With history of diagnosed NPC (n=9)	Group II: Without history of NPC (n=26)	p-value
WHO **Grading**			
WHO I	1^†^(11.1)	0 (0.0)	0.091
WHO II	3 (33.3)	11 (42.3)	0.640
WHO III	5 (55.6)	15 (57.7)	0.914

† This case was excluded from further analysis in table 4 because of the low number of patients in group WHO I.

**Table 4:** Correlation of outcome in 23 patients with NPC who had available data from both groups combined in relation to their WHO grading

WHO Grading	Outcome		p-value
	Alive (n=14)	Dead (n=9)	
WHO II (n=8)	3 (37.5)	5 (62.5)	0.094
WHO III (n=15)	11 (73.3)	4 (26.7)	

## DISCUSSION

NPC is an adult tumor more commonly encountered in the Chinese population than in the Caucasian population^(1,27)^. It is the most common type of tumor in the nasopharynx and is usually diagnosed relatively late because of nonspecific mild localizing symptoms and infrequent constitutional symptoms of malignancy in the tumor’s early stages^(2)^. One of the major ophthalmic--related presentations is cranial nerve palsies, especially when affecting the sixth cranial nerve, resulting in diplopia^(2)^. The tumor can rarely reach the orbit, causing proptosis by several routes, of which the most common is via the pterygopalatine fossa followed by the inferior orbital fissure or through the adjacent sinuses^(28)^. Therefore, this tumor is not commonly seen in ophthalmic practice. In a recent large study on 110 orbital lesions in adults in two tertiary eye centers, NPC was diagnosed as an orbital lesion in only 2 patients^(29)^. Following this, Alrashed et al. specifically reported their experience with NPC patients complaining of variable clinical manifestations who presented to ophthalmologists for the first time^(5)^.

Considering the possible aggressive nature of this tumor with local infiltrative features, a high level of suspicion and appropriate imaging techniques using magnetic resonance imaging are essential for assessing and staging NPC according to the latest 8th American Joint Commission on Cancer (AJCC) staging system^(28-30)^.

Considering that ophthalmologists might unusually be the first-line physicians facing patients with such a serious neoplasm, we decided to conduct this first systematic review and meta-analysis targeting this unique tumor in relation to ophthalmic presentations. Our aim was to highlight any variation in the demographics, clinical presentation, histopathological grading, and outcome that might be peculiar to NPC cases presenting to ophthalmologists. In 2021, Chang et al. reported the epidemiological aspects of NPC. They reported that the age of the patients ranged from 35 to 79 years, with a predominance of the tumor (triple) among men^(27)^. In our analysis, the overall age ranged from 22 to 92 years, with a male-to-female ratio of approximately 3:1, which is comparable with the report by Chang et al. In our analysis, we found that the demographics did not differ much between the two study groups under study. It is worth mentioning, however, that the group with initial ophthalmic presentation had a relatively wider and shifted age range, reaching 27-92 years compared with 22-74 years in the first group. Although this was not statistically significant, we advise ophthalmologists to be more suspicious when encountering older patients in their 90s who present with proptosis or unexplained nonocular visual disturbance.

With regard to the clinical presentation, it seems that the loss of vision observed in about half of the patients without a history of NPC is what brought these patients to the ophthalmic clinic relatively early, and it was the most common presentation in that group. The most common presentations of patients with prior diagnosis of NPC were diplopia and proptosis, followed by visual disturbance. This might indicate long-standing infiltrative tumors with higher chances for extensive involvement, causing cranial neve palsies, diplopia, and proptosis due to orbital extension, all of which appear relatively late. Other less common ocular manifestations include eyelid swelling or mass, orbital and/or facial pain, ptosis, Horner syndrome, and optic nerve swelling or pallor. The latter was uniquely found in group II only and might be an additional reason behind the visual loss in these patients. Most of these presentations are due to nerve involvement by the tumor or the mass effect of direct extension of the tumor. Ophthalmologists should keep these variations in clinical presentation in mind, even though none were significantly prevalent in one group over the other.

We assumed that group II patients with late onset of NPC and relatively earlier diagnosis would harbor tumors with better WHO classification. Almost all NPC tumors in both groups were of WHO II and III, with no statistically significant difference between the two groups. In 2016, Wang et al. highlighted the limited prognostic value of the WHO classification in NPC and proposed their own histopathological classification^(31)^. Others have recently validated the latest 8th AJCC staging system with a superior prognostic value, especially in the era of the treatment modality using intensity modulated radiotherapy^(30,31)^. Our analysis supports the possible limited importance of the WHO classification as a prognostic indicator. However, this is considered to be an observation rather than a solid general conclusion due to the sparse histopathological details in most of the collected literature and the fact that we were focusing on only the NPC cases that had ophthalmic manifestations, which belong to a specific subgroup of all reported NPC cases. In our review, the only statistically significant difference between the two groups was the outcome with a significantly higher proportion of patients who are alive (81% in group II compared with 29% in group I) within a comparable average follow-up period of 26.9 and 24 months, respectively (p=0.003). Our explanation is that visual disturbance aids in bringing these patients to the ophthalmologist earlier, before significant growth and invasion of the tumor are enough to cause proptosis and/or cranial nerve involvement with diplopia, which was more commonly encountered in group I patients with NPC.

There are several limitations to our study. We specifically targeted patients with NPC who had associated ophthalmic presentations before or following their diagnosis of NPC. Many published articles had insufficient data needed for our review, such as histopathological details, WHO classification, and outcome. EBV as a known risk factor for NPC was also not included in our data because of deficient reporting.

In conclusion, this systematic review has allowed us to conclude that there are similar demographics between patients with NPC who developed ocular problems following their diagnosis and patients with NPC who presented initially with ocular abnormalities mainly concerning their vision. NPC occurs within a very wide range of patient age, with a mean of 53 years, and it is generally three times more common in men. The ophthalmic clinical presentation is variable, and it is more likely for patients who are previously diagnosed with NPC to present with diplopia and/or proptosis along the course of their tumor progression. WHO grading did not correlate well with the general outcome of patients in both groups; thus, its prognostic value is questionable. Ophthalmologists might play a positive role in the early diagnosis of NPC when patients present to them initially complaining of visual disturbance. Further larger meta-analyses of NPC are warranted to investigate the risks, treatment benefits, and prognosis.
